# Impact of embryo quality and endometrial thickness on implantation in natural cycle IVF

**DOI:** 10.1007/s00404-020-05507-4

**Published:** 2020-03-24

**Authors:** Vlatka Tomic, Miro Kasum, Katarina Vucic

**Affiliations:** 1 Department of Obstetrics and Gynecology, Paracelsus Medical University, Müllner Hauptstrasse 48, 5020 Salzburg, Austria; 2 Department of Obstetrics and Gynecology, School of Medicine, University Hospital Centre Zagreb, University of Zagreb, Zagreb, Croatia; 3 European Medicines Agency, Amsterdam, The Netherlands

**Keywords:** Embryo, Endometrial thickness, Pregnancy rate, Natural cycle IVF, Prognostic factors

## Abstract

**Purpose:**

The aim of this study is to assess the effect of the endometrial thickness and embryo quality on the implantation potential in natural cycle IVF (NC-IVF).

**Methods:**

A retrospective single-center study was performed on 552 single embryo transfers after NC-IVF. The ‘quality' of the embryos was evaluated trough the number and regularity of blastomeres, degree of fragmentation, and nuclear content of cells. Endometrial thickness was measured in millimeters with transvaginal ultrasound on the day of hCG application.

**Results:**

Our findings showed a statistically significant difference in successful implantation until a plateau of 10 mm is reached (*p* = 0.001). Only one pregnancy was achieved where endometrial thickness was less than 7 mm, and this resulted in an early miscarriage. The predictors of favorable implantation were fragmentation (≤ 10%, *p* < 0.05) and the number of blastomeres (preferably 8-cell, *p* < 0.01) on day 3. Embryo quality (*R* = 0.052) and endometrial thickness (*R* = 0.18) were closely related to pregnancy rate. The overall implantation rate per embryo transfer was 18.8%.

**Conclusions:**

Embryo quality and endometrial thickness have a significant impact on implantation in NC-IVF. Highest implantation potential has an 8-cell embryo with ≤ 10% fragmentation in the third day following oocyte retrieval. Endometrial thickness of at least 7 mm seems to be the optimal edge of successful pregnancy.

## Introduction

Natural cycle IVF (NC-IVF) is a safe, low-cost, and patient friendly alternative to conventional controlled ovarian stimulation (COS). Those patients that could benefit from NC-IVF include those with a poor ovarian response, with a history of previous ovarian hyperstimulation syndrome (OHSS), and subfertile couples [1, 2]. Furthermore, without hormonal stimulation drug-induced side effects could be avoided. Patients who would prefer to have a more natural approach for personal, ethical, and religious reasons may also find natural IVF a suitable solution.

Endometrial receptivity and embryo quality are important factors of successful outcomes in stimulated IVF cycles [3].

Endometrial receptivity is a result of effects provided by ovarian steroid hormones, and is synchronized with fertilization and embryo development [4]. Extremely deviant endometrial morphology on the day of oocyte retrieval in stimulated IVF cycles with embryo transfer (ET) has been associated with deleterious effects on clinical pregnancy [5]. Measurement of the endometrial thickness by ultrasound is a simple, non-invasive method that has been studied as an indicator of endometrial receptivity, and a possible predictor of the potential success of IVF-ET treatment.

Findings are, however, inconclusive due to different induction protocols in use, limited sample sizes and various inclusion criteria [6–9]. Embryo quality is primarily assessed through an embryo grading system where embryos are graded according to their morphological appearance and are given a score that relates to their development at the time of assessment [10]. The goal is to provide a more precise estimate of implantation potential [11].

In contrast to a stimulated IVF cycle, in NC-IVF the treatment target is one follicle that spontaneously develops to dominance, which results in most of the cases with availability of single embryo for transfer. Conventional IVF-ET and three NC‐IVF treatments lead to a similar cumulative pregnancy rate, but the cost per pregnancy is 15% lower in NC-IVF [12, 13]. In addition, the embryo quality seems more favorable in NC-IVF when compared with conventional IVF [14]. A study from 2018 by von Wolff et al. [15] found thin endometrium as an independent negative prognostic factor in NC-IVF.

NC-IVF provides a unique opportunity to study and relate embryo characteristics to their implantation potential, without any selection or biases. The aim of our study is to assess the effect of endometrial thickness and embryo quality on the implantation potential in NC-IVF.

### Patients and methods

The study included 552 cases of ET after NC-IVF performed between 2010 and 2017 at University Hospital Center Sisters of Mercy, Zagreb, Croatia. Our study was exempt from the Institutional Review Board approval since it was a retrospective review of data already collected anonymously.

Inclusion criteria were as follows: patients aged < 38 years, BMI ≤ 25 m^2^/kg and one available embryo for transfer in a non-stimulated cycle where only human chorionic gonadotropin (hCG; Ovitrelle, Serono, Geneva, Switzerland) for triggering ovulation was used. No drugs were administered to prevent premature luteinizing hormone (LH) surge and possible rupture of the follicules. Serum concentrations of estradiol, progesterone, follicle stimulating hormone (FSH), and LH in serum were measured at the start of monitoring (first to fourth day of the cycle). On subsequent regular control visits estradiol, progesterone and LH were measured every 2 days, followed by ultrasound measurement of the follicle and endometrium thickness in millimeters (mm). By ascending slope of LH (> 20 mIU/mL) with decreasing estradiol levels and progesterone ≥ 1.5 ng/mL, the NC-IVF cycle was cancelled. Although monitoring for NC-IVF in our center is intensive, the cancellation rate was 12.4%. When the leading follicle was sized 18–20 mm, hCG was applied and the aspiration was performed 36 h later with the oocyte inseminated with motile spermatozoa approximately 2–5 h later. Fertilization was assessed 20 h later by determination of the number of pronuclei. The total rate of NC-IVF cycles with retrieved oocytes was 77.2%, and the fertilization rate was 82.4% with embryos scored 48 and 72 h after insemination.

The ‘quality’ of the embryos was evaluated through the number and regularity of blastomeres, degree of fragmentation, and nuclear content of cells. Embryos with more than two pronuclei and fragmentation of ≥ 50% were discarded for transfer.

Embryo transfer was performed 72 h after oocyte retrieval using a Cook soft catheter (K-soft 5100, Cook, Brisbane, Australia).

Measurement of the greatest endometrial thickness in the longitudinal plane of the uterus in transvaginal ultrasound (Voluson S6 and Voluson S8, RIC 5–9W, GE Healthcare) was measured from one endometrial–myometrium interphase to the corresponding interphase. The measurement recorded on the day of hCG administration was used as the referral value in this study. Pregnancy was first assessed by a positive hCG test 2 weeks after the oocyte retrieval and was confirmed by ultrasound visualization of intrauterine gestational sac with an embryo with heartbeats at 6 weeks gestational age.

### Statistical analysis

Statistical analysis was performed with SPSS 18.0. software (SPSS, Inc., Chicago, IL). A * χ*^2^ test was used to compare differences in patient and cycle characteristics and endometrial thickness on the day of hCG administration between pregnant and non-pregnant women. Logistic regression analysis was applied to determinate the influence of specific embryo characteristics on implantation. After univariate analysis, factors with *p* < 0.25 were retained for multivariate analysis. Multiple logistical regression analysis was used to assess the impact of endometrial thickness and embryo quality on outcome of NC-IVF. *p* < 0.05 was considered statistically significant.

## Results

The patients are divided into two groups, pregnant or non-pregnant women based on the β-hCG pregnancy test 2 weeks after ET and confirmed by transvaginal ultrasound examination at 6 weeks of gestation if the gestational sac with an embryo with heartbeats was present.

Patient and cycle characteristics are presented in Table 1. Age, body mass index (BMI), number of oocyte retrieved, number of embryos transferred, and estradiol serum level at hCG trigger showed comparable results between two groups. Embryo quality and endometrial thickness (11.4 mm ± 2.7 mm vs. 8.6 mm ± 2.3 mm) were higher in pregnant women than in non-pregnant (*p* < 0.05).

**Table 1:** Patient and cycle characteristics

	Pregnant women, *N* = 104	Non-pregnant women, *N* = 448	*p* value
Age (years)	33.2 ± 3.8	34.1 ± 3.3	NS
BMI (m^2^/kg)	24.4 ± 2.6	24.1 ± 3.4	NS
Endometrial thickness (mm)	11.4 ± 2.7	8.6 ± 2.3	< 0.05
Number of oocytes retrieved	1.1 ± 0.2	1.1 ± 0.1	NS
Number of embryos transferred	1.1 ± 0.1	1.0 ± 0.1	NS
Top quality embryos	0.4 ± 0.1	0.3 ± 0.2	< 0.05
Estradiol at hCG trigger (pg/mL)	268 ± 52	246 ± 74	NS

*NS* = non-significant

Pregnancy rate and endometrial thickness on day of hCG administration are shown in Table 2. Only one pregnancy was achieved with endometrium thickness less than 7 mm and this resulted in early miscarriage (Table 2). The nine miscarriages were registered in the group of pregnant women with endometrial thickness of ≥ 7 mm. Overall miscarriage rate was 9.6%. Our results indicate a statistically significant difference in successful implantation until a plateau of 10 mm is reached (*p* = 0.0011). After cutoff value of 10 mm, the difference in endometrial thickness between pregnant and nonpregnant women was not statistically significant.

**Table 2:** Pregnancy rate and endometrial thickness on day of hCG application

Endometrial thickness	Pregnant women	Non-pregnant women	*p* value
≤ 6 mm	1	71	< 0.0001
7 mm	4	64	< 0.0001
8 mm	6	124	< 0.0001
9 mm	11	70	< 0.0001
10 mm	15	39	0.0011
11 mm	16	21	NS
12 mm	20	24	NS
13 mm	14	13	NS
14 mm	11	12	NS
15 mm	3	6	NS
16 mm	2	3	NS
≥ 17 mm	1	1	NS
Total 552	104 (18.8%)	448 (81.2%)	

*NS* = non-significant

The implantation rate was 18.8% (104/552) per embryo transfer and clinical pregnancy rate was 17.03% (94/552). The birth rate was not analyzed in this study. According to the embryo quality, the highest implantation rate was found with 8-cell embryos on day 3, having ≤ 10% fragmentation, symmetrical blastomeres, and an absence of multinucleated blastomeres, i.e. so-called “top quality embryos”. In univariate and multivariate analyses, embryos with fragmentation less or equal to 10% showed the highest implantation potential and in utero survival (*p* < 0.01 and *p* < 0.05, respectively). Day 3 fragmentation of ≤ 20% also presented as a marker of implantation success in NC-IVF versus embryos with > 20% fragmentation (Table 3).

**Table 3:** Impact of embryo quality on implantation rate–regression analysis

	Univariate analysis		Multivariate analysis	
	^a^OR	*p* value	OR adj	*p* value
Embryo characteristics				
Blastomeres number day 2 (≥ 3 vs. 2)	1.70	^a^NS	1.62	NS
Blastomeres number day 3 (8 vs. 3–7)	2.48	< 0.01	2.18	< 0.01
Fragmentation day 3 (≤ 10% vs. > 10%)	1.93	< 0.01	1.71	< 0.05
Fragmentation day 3 (≤ 20% vs. > 20%)	1.78	< 0.05	1.68	< 0.05
Multinuclear present (never vs. ever)	1.54	NS	1.46	NS
Blastomeres shape (symmetrical vs. non-symmetrical)	1.61	NS	1.48	NS

^a^*NS* non-significant, *OR* odds ratio

Blastomere numbers on day 3, but not on the day 2, showed predictive values (*p* < 0.01) for ongoing pregnancy. The overall implantation rate per embryo transfer was 18.8%.

Influence of endometrial thickness and embryo quality on implantation potential suggests their independent effects on pregnancy rate when analyzed by multiple regression analysis (Table 4).

**Table 4:** The relationship between endometrial thickness and embryo quality on IVF outcome–multiple logistical regression analysis

Independent variable	Endometrial thickness	Embryo quality (top)
Regression coefficient	0.18	0.052
*p* value	< 0.05	< 0.01

## Discussion

The embryo characteristics identified as the most optimal (8 cells on day 3 with less or equal to 10% of fragmentation) corresponded, as expected, with highest pregnancy rate in NC-IVF in this study.

The administration of gonadotropins in stimulated IVF cycles may influence the cleavage capacity of embryos, although Ziebe et al. [16] found comparable embryo quality and cleavage stages between natural and stimulated cycles. The receptivity of the endometrium may be adversely affected by ovarian hyperstimulation, leading to a discordant maturation between the embryo and the endometrium which may result in failed implantation [17]. In vitro studies have revealed novel roles for the decidualized endometrium as a biosensor of embryo quality, with the embryo itself responsible for ~ 30% of implantation failures, whereas inadequate uterine receptivity is responsible for approximately 70% of implantation failures [18]. Furthermore, higher endometrial thickness observed in stimulated cycles may be related with more mature endometrium that favors embryos with higher number of blastomeres, i.e. in more advanced stage of development.

Endometrial characteristics in natural cycle IVF are considerably different from those in medically induced cycles [19]. In NC-IVF two cells embryos in day 2 without any other abnormalities were found to have good implantation potential.

Multivariate analysis showed that blastomere numbers of at least two did not influence their implantation potential. Pelinck et al. [20] suggested that the implantation potential of embryos with a low number of blastomeres on day 2 is underestimated, which was confirmed by findings of our study. Blastomere numbers on day 3 had a strong predictive value of their implantation potential, underpinning 8-cell as a significant factor in clinically successful pregnancy. Fragmentation is the other significant factor. An almost twofold increase in ongoing pregnancy rates were observed for fragmentation up to 10% in our results. Even fragmentation of ≤ 20% presented with similar OR when compared with fragmentation > 20%. When compared with NC follicles, putative markers associated with oocyte quality and follicle maturation were significantly different from those in gonadotropin-stimulated follicles [21]. Therefore, it is unclear whether the results of our study apply to embryos derived from COS, although similar findings are observed in the previous COS-IVF studies [22, 23]. One study revealed a significant negative impact of multinucleation on implantation and birth in COS-IVF [24], whereas our study failed to demonstrate such a relationship in NC-IVF.

In our study, endometrial thickness of at least 7 mm is found to be the optimal margin for successful implantation in NC-IVF. Conflicting results exist concerning the possible relationship between endometrial thickness and treatment outcome after IVF [3, 6–8, 25]. The majority of previous studies reported small series (less than 200 cycles) or used different ovarian stimulation protocols. Systematic review and meta-analysis, although limited with heterogenity of study data, showed that pregnancy rates improved with increasing endometrial thickness, but pregnancy rates still appeared to be significantly lower up to an endometrial thickness value of ≤ 10 mm [26]. The authors do not recommend the use of endometrial thickness as a tool to decide on cycle cancellation. The differences that induction protocols and/or multiple embryos may bring are overcome in NC-IVF, which may serve as the best model for investigating the possible link between embryo quality, endometrial thickness, and implantation success.

The endometrial thickness threshold established in our finding is 7 mm, below which pregnancy rate decreased rapidly. More than 50% of pregnancies with endometrial thickness below 7 mm resulted in spontaneous miscarriage [8, 16, 27]. A study of Al-Ghamdi et al. [28] demonstrated a significant increase in the pregnancy rate with increasing endometrial thickness, even independent of the quality of embryos transferred. Their conclusion was confirmed in a recent study of Liu et al. [29]. However, direct comparison between NC-IVF and conventional IVF is hard, since hormonal milieu and treatment form differ significantly. The only NC-IVF study by von Wolff et al. [15] showed reduced pregnancy rate with an endometrial thickness ≤ 7 mm, but in contrast to conventional IVF a tendency to lower pregnancy rate with thick endometria (> 11 mm). In contrast, in our study after cut-off value of 10 mm, the difference in endometrial thickness between pregnant and non-pregnant women was not statistically significant.

In our study, lower endometrial thickness (but not lower than 7 mm) and high embryo quality were predictors of successful pregnancy and vice versa. It seems reasonable to believe that to some extent higher endometrial thickness could compensate the poorer embryo quality and vice-versa, but with embryo quality below optimum (≤ 20% fragmentation, 6–8 cells on day 3) and endometrial thickness below 7 mm the chance of successful pregnancy is poor.

Our study is, to our best knowledge, the first large homogenous cohort of NC-IVF investigating and connecting embryo quality and endometrial thickness. Nevertheless, some limitations have to be discussed, such as retrospective design and therefore chance of biases as well as results limited to women below 38 years of age and natural cycle IVF.

## Conclusion

Embryo quality and endometrial thickness have significant impact on implantation in NC-IVF. Highest implantation potential has 8-cell embryos with less or equal to 10% fragmentation on third day following oocyte retrieval. Endometrial thickness of at least 7 mm seems to be the optimal edge of successful pregnancy.
